# Ancient DNA reveals the chronology of walrus ivory trade from Norse Greenland

**DOI:** 10.1098/rspb.2018.0978

**Published:** 2018-08-08

**Authors:** Bastiaan Star, James H. Barrett, Agata T. Gondek, Sanne Boessenkool

**Affiliations:** 1 Centre for Ecological and Evolutionary Synthesis, Department of Biosciences, University of Oslo, PO Box 1066, Blindern, 0316 Oslo, Norway; 2 McDonald Institute for Archaeological Research, Department of Archaeology, University of Cambridge, Downing Street, Cambridge CB2 3ER, UK

**Keywords:** high-throughput sequencing, Viking Age, Middle Ages, aDNA, *Odobenus rosmarus rosmarus*

## Abstract

The importance of the Atlantic walrus ivory trade for the colonization, peak, and collapse of the medieval Norse colonies on Greenland has been extensively debated. Nevertheless, no studies have directly traced medieval European ivory back to distinct Arctic populations of walrus. Analysing the entire mitogenomes of 37 archaeological specimens from Europe, Svalbard, and Greenland, we here discover that Atlantic walrus comprises two monophyletic mitochondrial (MT) clades, which diverged between 23 400 and 251 120 years ago. Our improved genomic resolution allows us to reinterpret the geographical distribution of partial MT data from 306 modern and nineteenth-century specimens, finding that one of these clades was exclusively accessible to Greenlanders. With this discovery, we ascertain the biological origin of 23 archaeological specimens from Europe (most dated between 900 and 1400 CE). These results reveal a significant shift in trade from an early, predominantly eastern source towards a near exclusive representation of Greenland ivory. Our study provides empirical evidence for how this remote Arctic resource was progressively integrated into a medieval pan-European trade network, contributing to both the resilience and vulnerability of Norse Greenland society.

## 1. Introduction

Atlantic walrus (*Odobenus rosmarus rosmarus*) has long been exploited for its ivory, which was a popular material for the manufacture of luxury art objects in medieval Europe. With isolated earlier and later exceptions, its use started in the tenth century, peaked with the Romanesque art style of the twelfth century, and subsequently declined [1–4]. To what extent this European demand for ivory encouraged exploration and colonization of the North Atlantic region and the Arctic has been extensively discussed [5–9]. While the Atlantic walrus is widely distributed—with populations being found from Siberia to Canada [10]—of all potential sources it is *Norse Greenland* for which ivory trade has been suggested as especially important. First, the initial exploration and settlement of Greenland *ca* 980–990 CE has been attributed to the hunt for ivory [7]. Second, the thirteenth- and early fourteenth-century peak in transatlantic trade to Greenland, and architectural (particularly church) investments there, have been similarly connected with the walrus [6,9,11–14]. Finally, the abandonment of the Norse colony—its Western Settlement in the fourteenth century and its (more southern) Eastern Settlement in the fifteenth century—has been blamed on the declining popularity of walrus ivory in Europe and/or on a switch to alternative Arctic sources such as Svalbard or Russia [7,15]. However, no studies have directly traced medieval European ivory back to distinct walrus populations in order to evaluate the importance of various Arctic sources between the tenth and fifteenth century CE.

The hunting of walruses for ivory by Norse Greenlanders is testified by archaeological finds of skulls, skull fragments, tusk offcuts, cheek teeth, and carved objects [9,16,17]. Post-cranial walrus bones (that represent hunting for meat rather than ivory) are rare from Norse sites in Greenland, particularly from the Eastern Settlement. Based on historical sources, most hunting took place further north along the west coast, mainly around Disko Bay, which can explain the lack of post-cranial bones at the settlements [9,18–21]. The actual transport of walrus products—ivory, hide ropes, and even a decorated walrus skull—from Greenland to Europe as gifts, tithes, and trade goods is also described in historical sources. Although these sources vary in their historicity and are dated to the thirteenth century CE or later, they describe practices thought (by their medieval authors and modern scholars) to have had an earlier origin [6,7,13,21]. Yet another major source of ivory was the Barents Sea region of Arctic Fennoscandia and Russia. This eastern source was documented as early as the late ninth century CE, when the Arctic Norwegian chieftain Ohthere visited the court of King Alfred of Wessex in England [22,23]. The continued importance of this source is implied by the great abundance of walrus ivory known from medieval Novgorod—an important trading town with an extensive network into Arctic Fennoscandia and Russia [24,25]—and by the hunting of the Arctic European walrus in the Svalbard Archipelago between the sixteenth and twentieth centuries [26]. Finally, walruses were also initially hunted in Iceland during its colonization in the late ninth and tenth centuries [7,9,27], but by the twelfth–thirteenth centuries, when the island's earliest laws and narrative texts were first recorded, Icelandic walruses were reduced to isolated visitors only [9,28]. Isotopic analyses indicate that Icelandic ivory finds dated to the Viking Age were probably from local hunting [7], but there exists no empirical evidence on the geographical origin of the ivory imported to European trading centres such as Trondheim, Bergen, Oslo, Dublin, London, Sigtuna, and Schleswig during the chronology of the Norse Greenland settlements. Here, we fill this knowledge gap.

Genetic analyses of modern walruses reveal significant population structure based on microsatellite variation [29–32], mitochondrial (MT) restriction fragment length polymorphism (RFLP) [29,30], and partial MT sequence variation [33], which agrees with high levels of observed site fidelity [34,35]. Analyses of RFLP data reveal the widespread distribution of a common MT haplotype across the entire North Atlantic region, with a small subset of haplotypes that is geographically restricted to populations in western Greenland and the Canadian Arctic [29,30]. So far, the limited resolution of the available MT data has made it difficult to determine whether this distinct distribution is the result from pre- or post-glacial isolation [29,30,33,36,37].

Here, we analyse the entire mitogenomes of 20 archaeological walrus rostrums and three tusk offcuts traded to western Europe to determine their geographical origin. Although aDNA can trace such specimens towards their biological source [38–40], it is difficult to apply this destructive technique to objects of fine art such as worked ivory. Nonetheless, partial walrus skulls (rostrums) with *in situ* tusks were *also* transported to Europe [41]. In rare instances these ‘ivory packages’ remain intact, while in others they were broken up to extract the ivory [41–46]. These rostrums and rostrum fragments can serve as proxies for the ivory they carried and can be sampled for biomolecular analyses without the need to damage ivory artefacts. We include mitogenomes from a set of controls—representing samples whereby hunting location can be confidently assumed—consisting of four medieval samples from Greenland and 10 samples from eighteenth and nineteenth-century Svalbard. We compare our data to 306 modern and nineteenth-century specimens from the Barents Sea region, Greenland, and the Canadian Arctic [32,33,36,37] to infer the geographical origin of European walrus imports during the chronology of the Norse occupation of Greenland.

## 2. Material and methods

### (a) Sampling

**Figure 1.**
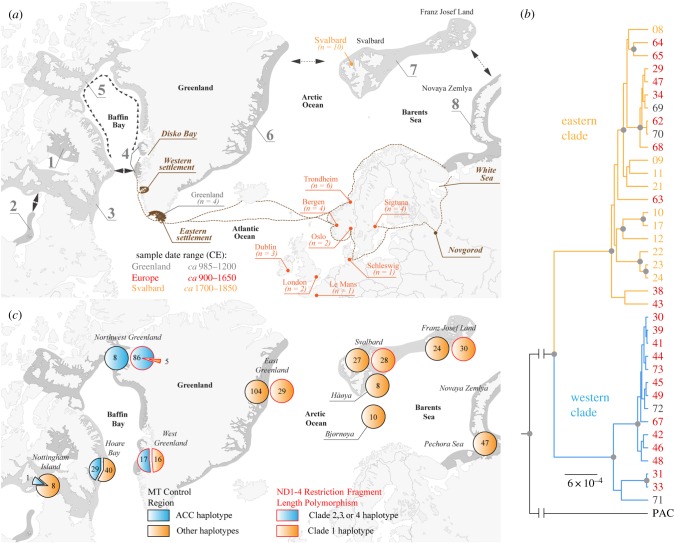
(*a*) Population distribution, potential trade routes, and sample locations of Atlantic walrus in the northern Atlantic region. The range of modern Atlantic walrus (*dark grey*) and dispersal routes (*black arrows*) follow [47] and [31]. Eight breeding populations are recognized [47]; (1) Foxe Basin, (2) Hudson Bay, (3) Hudson Strait, (4) West Greenland, (5) North Water, (6) East Greenland, (7) Svalbard/Franz Josef Land, (8) Novaya Zemlya. Trade routes from Greenland—including the location of Norse settlements—and northern Fennoscandia/Russia (*brown*) indicate possible sources from which walrus ivory was exported during the Middle Ages. The Svalbard specimens (*orange*) were originally from hunting stations of the 1700s and 1800s. The other Atlantic walrus specimens (*red*, *grey*) were obtained from museum collections. (*b*) Bayesian phylogenetic tree obtained using BEAST [48] based on 346 mitochondrial single nucleotide polymorphisms (SNPs) using Pacific walrus (PAC) as an outgroup. Numbers represent the different specimens listed in electronic supplementary material, table S1. The colour of the numbers (*grey*, *red*, *orange*) match the sampling locations as in (*a*). Branches with a posterior probability of one (*grey circles*) are indicated. (*c*) Distribution of RFLP and control region (CR) haplotypes of modern Atlantic walrus. The RFLP clade classification follows [30]. The distribution of a distinct ACC CR haplotype is from 306 specimens (see Methods).

We sampled 37, morphologically identified walrus bone and ivory specimens from western Europe (*n* = 23), Greenland (*n* = 4), and Svalbard (*n* = 10, figure 1*a*; electronic supplementary material, table S1). Specimens have been dated by archaeological context, associated artefacts, or (in one case) a runic inscription. Direct radiocarbon dating was not used because precise marine reservoir correction requires a Δ*R* value which cannot be predicted without catch location [49]. Two late outliers that post-date the Norse occupation of Greenland are included for comparison, one from a context dating 1500–1532 CE and one post-dating 1600 CE. Two other samples are undated, being from old excavations, but are probably medieval in origin. The specimens were originally obtained from excavations and subsequently archived in museum collections, although in Le Mans a rostrum with a thirteenth–fourteenth century runic inscription on one of its tusks has come down to posterity intact [41,50]. The Le Mans rostrum must have been kept as a display piece over the centuries, until being acquired by Musée Vert, muséum d'histoire naturelle du Mans [41]. The set of control samples consisted of four medieval rostrums, excavated at the site of Igaliku (Gardar) in Greenland's former Eastern Settlement [51], and 10 specimens from eighteenth- and nineteenth-century Svalbard that were dated based on the documented use of the hunting stations from which they were collected.

### (b) DNA extraction, library creation, and sequencing

All DNA extraction and pre-PCR protocols were performed in a dedicated laboratory at the University of Oslo following strict aDNA precautions [52,53]. Samples were exposed to UV (10 min) on each side (total dosage of 4 800 J m^−2^) before cutting. Cut fragments were again exposed to UV (10 min) on each side before milling using a custom-designed stainless-steel mortar [54] or a Retsch MM400 mill. Extraction used a bleach and pre-digestion protocol [55], apart from the Greenland samples for which only pre-digestion was used [56]. Bleach washes were done in duplicate (150–200 mg of powder each) [55], followed by pre-digestion and an overnight, second digestion [56]. The two eluates were combined and concentrated (Amicon-30kDA Centrifugal Filter Units), extracting DNA using Minelute (Qiagen) according to the manufacturer's instructions. DNA was eluted in 60 µl pre-heated (60°C) elution buffer (EB), incubating for 15 min at 37°C [57]. Negative controls were included in all extraction experiments. Blunt-end Illumina libraries [39,58] were amplified with sample-specific 7 bp P7 indexes in triplicate, 25 µl reactions [40], quantified (Bioanalyzer 2100, Agilent), sequenced (125 bp paired-end, Illumina HiSeq 2500), and demultiplexed (--mismatch 0).

### (c) Data processing and analyses

Sequencing reads were processed (PALEOMIX [59]), collapsed (AdapterRemoval v1.5 [60]), and aligned to the Pacific walrus nuclear genome [61] and the Atlantic walrus mitogenome [62] (BWA *aln* v.0.7.5a-r405 [63]). Pacific walrus reads [61] were aligned to the Atlantic walrus mitogenome to obtain a Pacific walrus MT sequence (electronic supplementary material). aDNA damage was investigated (mapDamage v.2.0.6 [64]) excluding alignments with a MapQ-value < 25. Genotypes were jointly called (GATK v.3.4.46 [65] *Haplotypecaller --ploidy* 1 & *Genotypecaller*), after duplicate removal (PicardTools v.1.96), indel realignment (*IndelRealigner*), and filtered (BCFTOOLS v.1.6 [66] *-i* ‘FS < 60.0 && SOR < 4 && MQ > 30.0 && QD > 2.0’, *--SnpGap* 10). Indels were excluded and genotypes with quality less than 15 and read depth less than 3 were set as missing (VCFTOOLS v.0.1.14 [67]).

Phylogenetic analyses were performed (BEAST 2.4.7 [48]) with Pacific walrus as outgroup, implementing the Hasegawa, Kishino, and Yano (HKY) substitution model with the Yule tree prior and a strict clock (jModeltest2 v.0.1.10 [68]). Timing to most recent common ancestor (MRCA) was estimated using a faster rate of 0.75 × 10^−7^ substitutions/site/year from southern elephant seal control region (CR) data [69] and a slower rate of 0.7 × 10^−8^ estimated from baleen whale cytochrome *b* data [70,71], because the CR mutates relatively fast compared to other parts of the mitogenome [72]. The Markov chain Monte Carlo (MCMC) (10 million gen, pre-burnin 1 million gen) was sampled every 1 000 trees and effective sample size (ESS) values above 200 confirmed convergence (Tracer v.1.6.0 [73]). After a 10% burnin (TreeAnnotator) the maximum clade credibility tree was visualized (FigTree v.1.4.3). A haplotype genealogy was drawn using Fitchi [74]. Differentiation among lineages and clade supporting SNPs were obtained (*smartPCA* v.6.1.4 [75] --*snpweightoutname*; electronic supplementary material, figure S3).

We integrated our data from archaeological walrus specimens with data from modern walrus samples on MT RFLP variation and MT CR haplotypes. First, we investigated if observed genetic divergence in archaeological walruses can be detected in previously published RLFP data [29,30]. In such a case, it should be possible to identify specific SNPs that alter the sequence motifs of restriction fragment binding sites. Clade-specific modification of RFLP motifs was obtained using *FindMotif*, (IGV [76]; electronic supplementary material). Second, we compared the CR haplotypes of our archaeological specimens to CR data of modern and historic walrus populations. CR population data [32,33,36,37] were aligned to the Atlantic walrus MT genome and genotypes at position 15 564, 15 760, and 15 779 were scored (IGV [77]; electronic supplementary material, table S3). Finally, the binomial probability of observing different western and eastern clade ratios before and after *ca* 1125 CE was calculated by simulating variable contributions of a western Greenlandic/Canadian and Northeast Atlantic source, assuming 100% eastern clade specimens in the Northeast Atlantic populations and 48% in western Greenland/Canada (see below). Probabilities for source admixture were scaled to one for each period.

## 3. Results

### (a) Phylogeography of Atlantic walrus mitogenomes

We obtained 520 million paired sequencing reads for 37 samples that passed downstream filtering (figure 1*a*; electronic supplementary material, table S1). These samples contained 0.01 to 71% endogenous DNA and yielded 4.8 to 438-fold MT coverage (electronic supplementary material, table S2). The reads show the typical fragmentation and deamination patterns expected from post-mortem degradation (electronic supplementary material, figure S1). Including a *de novo* Pacific walrus MT sequence as outgroup (electronic supplementary material), a Bayesian phylogenetic analysis of 346 SNPs reveals two fully supported monophyletic clades in the archaeological Atlantic walrus data (figure 1*b*). The medieval walruses belong to either clade, whereas all the eighteenth and nineteenth century CE specimens from Svalbard fall into one clade. We tentatively define the clade containing the Svalbard samples as *eastern*, and the other clade as *western* (figure 1*b*). Of the four Greenland samples, two specimens fall into the western and two into the eastern clade (figure 1*b*). The two major lineages form distinct haplotype genealogies, with each clade separated by 21 or 18 substitutions from the most recent common ancestor (MRCA; electronic supplementary material, figure S2). Using two different substitution rates, the time to the MRCA was estimated to have been 23 400 (95% highest posterior density (HPD): 14 539–34 522) to 251 120 (95% HPD: 163 819–355 131) years ago.

A Principle Component Analysis (PCA) shows two significantly differentiated clusters supported by clade-specific SNPs that are located throughout the entire mitogenome (electronic supplementary material, figure S3). We investigated if these clade-specific SNPs can be associated with RFLP and control region (CR) data from modern (and historic) walrus populations. First, the RFLP studies focused on ND1, ND2, and ND3/4 MT gene regions [29,30]. We find 5 clade-specific SNPs in NADH dehydrogenase 1 (ND1), 3 in ND2, and 9 in ND3/4 (electronic supplementary material, figure S3*c*). At least one of these SNPs alters a RFLP motif depending on clade membership in *each* of these ND genes (electronic supplementary material). The combination of enzymes [29,30] can therefore separate the western and eastern clades we identify in our mitogenomes—on multiple restriction sites and within each ND gene region. While these RFLP analyses report four MT clades, the data show a pronounced bimodal divergence, with the majority of distinctive restriction sites and highest statistical support observed between clade 1 and any combination of clades 2–4 [29,30]. Clades 2–4 comprise 94% of the Northwest Greenland and 52% of the West Greenland population [30] (figure 1*c*). Crucially, these clade 2–4 haplotypes are exclusively identified in western Greenland and Artic Canada, and are absent in the Northeast Atlantic [29,30]. Clade 1 is found in every individual in the Northeast Atlantic and in various proportions in western Greenland (figure 1*c*).

Second, we investigated a 499 bp section (between 15 328 and 15 827 bp) of an extensive CR population dataset [32,33,36,37]. We observe three SNPs in this section, for which 12 out of 15 archaeological western clade specimens share a distinct A_15564_ C_15760_ C_15779_ haplotype (electronic supplementary material, figure S3*c*). Reanalysing the CR data for this ACC haplotype, we identified 38 out of 306 Atlantic walruses with the same haplotype (electronic supplementary material, table S3). The ACC haplotype is fixed in the Northwest Greenland population, co-occurs mixed with other haplotypes in Canada, yet is absent from the Northeast Atlantic (figure 1*c*). The distribution of this CR haplotype is therefore analogous to the RFLP distribution. We derive that the RFLP and CR analyses detect variation explained by the monophyletic lineages of our medieval and eighteenth and nineteenth century CE specimens. Moreover, these analyses reveal a consistent distribution, whereby a subset of haplotypes is geographically restricted to western Greenland and Canada. Since the ACC haplotype of our western clade is only found in western Greenland and Canada, we conclude that the European archaeological specimens belonging to this clade came from Norse Greenland, either because they were hunted by the Norse or because they were traded from further north and west via contact with indigenous Dorset or Thule peoples [18,19,21,78,79]. In contrast, haplotypes of the eastern clade may originate from either side of the Atlantic Ocean (figure 1*c*).

### (b) The chronology of historic walrus trade

**Figure 2.**
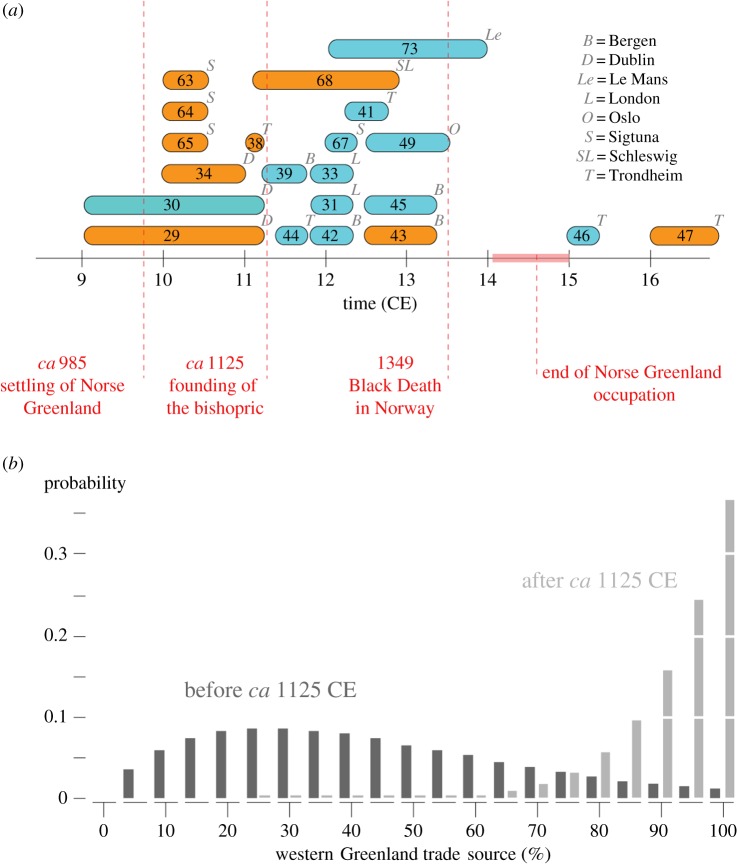
Chronology of Atlantic walrus specimens in Europe. (*a*) Archaeological specimens classified as western clade (*blue*) or eastern clade (*orange*) are plotted according to their estimated age range. The start and end of the Norse Greenland occupation, the founding of the bishopric, and the arrival of the Black Death in Norway are indicated (*dashed red lines*). For each specimen, its location (*light grey*) is indicated. (*b*) Probability of obtaining the observed sample of eastern and western clade specimens as a function of a variable contribution of a western Greenland source. The probability was calculated for those samples obtained before (*dark grey*) or after (*light grey*) the founding of the Greenland bishopric, excluding the sixteenth and seventeenth century CE specimens.

The dates of the archaeological walrus specimens cover the entire chronology of the Norse Greenland occupation, with two outliers post-dating its abandonment (figure 2*a*; electronic supplementary material, table S3). Before the founding of Greenland's bishopric in the 1120s CE [80]—during the settlement phase and the early period of Norse occupation—we identified one western and six eastern clade specimens in Europe (figure 2*a*). In contrast, during the later occupation, between the 1120s and the end of the fourteenth century, we observe 10 western and two eastern clade specimens. The timing of this significant increase (*p*-value = 0.0063, Fisher's exact test) in western clade specimens thus coincides with new ecclesiastical infrastructure in Greenland [80] and the height of Romanesque ivory carving in Europe [3]. We have not yet discovered European walrus rostrums that date specifically to the century of Norse Greenland's abandonment (the 1400s). By this date walrus ivory had long gone out of fashion, with the Gothic style using different materials such as elephant ivory [4]. Of the two later outliers in our dataset (both from Trondheim), one from the archbishop's palace is of the western clade, dated 1500–1532. It may represent redeposition of an earlier find, or the latest known export of a walrus rostrum from Greenland. The second outlier postdates 1600 CE and is of the eastern clade.

Based on RFLP data, eastern clade specimens make up nearly half (48%) of the modern West Greenland population—which is closest to the Disko Bay hunting grounds of the Greenland Norse (figure 1*c*). Albeit a small sample size, the medieval Greenland specimens from Gardar also have such a 50/50 distribution (figure 1*b*), suggesting long-term temporal stability of these haplotype frequencies and showing that eastern clade trade specimens could also have originated from Norse Greenland. We calculated the binomial probability of the observed ratio of western and eastern clade archaeological samples in the period *before* and *after* the founding of the Greenlandic bishopric *ca* 1125 CE (excluding the two outliers from Trondheim), given variable contributions from western Greenlandic/Canadian and Northeast Atlantic/European Arctic sources. This probability is calculated assuming that populations in the Northeast Atlantic are fixed for the eastern clade, whereas those in western Greenland and Canada comprised a mixture of clades, following the RFLP frequencies (48% eastern clade) of the West Greenland population. The probability distribution of the samples dated before the bishopric's founding shows evidence of geographical admixture, with a most likely contribution of a western source between 20% and 30% (figure 2*b*). In contrast, the samples dated after the founding and before *ca* 1400 CE show evidence for a near 100% western source (figure 2*b*). Thus, in its early period, it is statistically unlikely that Norse Greenland was an exclusive source of walrus for Europe. The Barents Sea region is the most likely alternative based on the Ohthere account of the late ninth century. Iceland and (less likely, due to difficult summer ice conditions) northeast Greenland are other possibilities [7,21]. In the later period between *ca* 1125 and *ca* 1400 CE, however, the eastern clade samples can—with high statistical probability—have come entirely from Norse Greenland together with the western clade specimens.

## 4. Discussion

We have reconstructed a chronology of long-distance ivory trade during the medieval period by investigating complete mitogenomes of archaeological Atlantic walrus specimens from Greenland, Svalbard, and Europe. Specifically, we distinguish whether individual walruses were obtained from a western Greenland source and our work has evolutionary, ecological, and archaeological implications.

We discover that Atlantic walrus comprises two major, monophyletic MT lineages. Several observations support a hypothesis that these lineages have evolved in glacial refugia on either side of the Atlantic Ocean. First, we estimate a divergence date between 23 400 and 251 120 years ago, well before or during the last glacial maximum (LGM). Second, (sub)fossil walrus bones dated over 10 k years BP have been found on both the European and the North American continent *at lower latitudes* compared to the walrus's modern distribution [81,82]. Third, glacial refugia on either side of the Atlantic have also been proposed to explain (partial) MT divergence in marine mammals with a similar coastal ecology like walrus, such as harbour seal (*Phoca vitulina*) [83], harbour porpoise (*Phocoena phocoena*) [84], and grey seal (*Halichoerus grypus*) [85]. Based on these observations, we conclude that the two MT lineages have evolved in spatial separation on either side of the Atlantic Ocean.

Such a scenario further explains the distinct geographical distribution of RFLP and CR population data, which show that haplotypes associated with the western clade occur solely in western Greenland and Canada [29–32,37]. The lack of such haplotypes in the Northeast Atlantic, despite the genetic analysis of hundreds of specimens covering multiple decades [29–32,37] indicates that gene flow has been asymmetrical, with eastern clade females dispersing to the western Atlantic but not *vice versa*. This dispersal follows the direction of the East Greenland Current [86], suggesting that ocean currents influence the tendency of walrus dispersal. Historically, a similar asymmetrical pattern has been observed on a regional scale in Baffin Bay—with walruses migrating counterclockwise, following the direction of the coastal currents and the breakup of sea ice [31,47]. Asymmetrical gene flow has also been observed between breeding areas of Pacific walrus in the Bering Sea [87], although the direction of ocean currents can be variable in this region [88]. Our study suggests that the direction of female dispersal from Eastern Greenland to Baffin Bay has been persistent since these two MT lineages came into secondary contact.

The possibility of geographical change in clade distribution between past and present populations is an inevitable challenge in aDNA studies (e.g. [89,90]). Nonetheless, it is highly improbable that western clade walruses could have occupied the historically recorded hunting grounds of the Barents Sea region in the Middle Ages, given the universal distribution of the eastern clade throughout the Northeast Atlantic region, the direction of the East Greenland current [86], the predominantly coastal habitat of the walrus [10], and the high levels of natal homing of walruses [30,34,35,91,92]. Moreover, given the location of Iceland well within the distribution of the eastern clade, and its recorded accessibility to Svalbard walruses [93], it is most likely that Icelandic walruses were of the eastern clade.

Thus, with our discovery of these two MT lineages and their geographical distribution, we provide quantitative, empirical evidence on the source of walrus imports to medieval Europe, and with that re-evaluate past hypotheses on the role of walrus ivory in the origins, efflorescence, and collapse of Norse Greenlandic society. Only one of our seven samples predating the 1120s CE is of the western clade. The other six samples of this period are of the eastern clade and could have come from a variety of sources, including Greenland, but it is probable that most derived from the Northeast Atlantic. Thus the theory that walrus ivory was a primary motive for the initial exploration and settlement of Greenland around 980–990 CE may need reconsideration [7]. Other relevant factors include the optimal conditions of the medieval climate anomaly, the search for new (pastoral) farmland, and the politics of exile noted in medieval Icelandic sources [7,94,95]. Conversely, 10 of 12 specimens dating between the 1120s and *ca* 1400 CE are of the western clade and must have arrived in Europe via Norse Greenland. Moreover, the observed ratio of eastern and western clade specimens has a high probablility of deriving exclusively from western Greenland. Thus, the documented heyday of Norse Greenlandic settlement, trade, and architectural elaboration (particularly evident in churches and their accoutrements)—between the twelfth and fourteenth centuries—did coincide with exports of walrus ivory. In fact, our data suggest that the Greenland trade of this commodity may have held a near monopoly in western Europe. The historically and/or archaeologically attested walrus hunts of the ninth–tenth centuries in the Barents Sea region and Iceland may have declined or ended by the early twelfth century, either because of rising Greenlandic exports or local reduction/extirpation of populations.

The reasons for the significant shift in trade cannot be inferred from aDNA alone and historically contingent local factors, as well as broader socioeconomic and environmental developments can be invoked as potential contributors. In thirteenth–fourteenth century Iceland it was believed that the Greenlanders used walrus products as gifts to influence the policies of Scandinavian monarchs, for example in the story of Einar Sokkason (*Grænlendinga tháttr*) [2]. Although the historicity of Einar's use of walrus ivory to secure a Greenland bishop and episcopal see in the early twelfth century cannot be confirmed, tithes (including papal dues) were paid in this material during the thirteenth and fourteenth centuries [1,6]. Thus both local initiative and the reach of pan-European church infrastructure potentially played a role in the naissance and maintenance of the Greenland ivory trade. Moreover, the eleventh to thirteenth centuries represent a period of major demographic and economic growth in Europe, in part due to environmental conditions favourable to cereal agriculture [96]. A growing urban and elite demand was served by transport from increasingly distant sources in a process of proto-globalization. Our discovery shows that Greenland was well integrated into this network.

Less can be said about the end of the Greenland colony based on the evidence here. Is the absence of sampled (or known) European finds of walrus rostrums from the fifteenth century evidence for the end of trade? Is the single, sixteenth-century western clade specimen an example of the last, isolated, Greenland export, or is it a redeposition of an earlier find? These are classic challenges of archaeological interpretation. Nonetheless, it is a conspicuous observation that Greenland may have been the exclusive supplier of walrus ivory to Europe between the 1120s and the fourteenth century. The demise of Norse Greenland would therefore have reduced European supplies of this raw material, whereas a decline in demand would have undermined Greenland's social and economic organization. Whatever other factors may have been influential—from the Little Ice Age [97–100], to gradual out-migration [99,100], to the impact of the Black Death (1346–1353) on European markets [2,96]—the cessation of trade in walrus ivory must have been significant for the end of Greenland's Eastern and Western settlements. Greenland is often discussed as a general example of both human resilience and vulnerability in the face of environmental and economic change [98,101–104]. Thus, the implications of this study—that the influence of ecological globalization for the Greenlandic Norse started small yet became paramount—extend far beyond medieval Europe.

## Supplementary Material

SI_aDNA_Greenland_Norse_walrus_ivory_trade

## Data accessibility

All ancient read data are available at the European Nucleotide Archive (ENA, www.ebi.ac.uk/ena) under study accession number PRJEB25536.
