# Supplementary material for: Ancient DNA reveals the chronology of walrus ivory trade from Norse Greenland
Source: Proc Biol Sci. 2018 Aug 8;285(1884):20180978. doi: 10.1098/rspb.2018.0978 (PMC6111184; doi:10.1098/rspb.2018.0978)
Supplement: SI_aDNA_Greenland_Norse_walrus_ivory_trade [file rspb20180978supp1.docx]

**Electronic Supplementary Information**

***Ancient DNA reveals the chronology of walrus ivory trade from Norse Greenland***

Bastiaan Star^1*†^, James H. Barrett^2*†^, Agata T. Gondek^1^, Sanne Boessenkool^1*^

^*^ *Corresponding author*

^†^ *These authors contributed equally*

*1 Centre for Ecological and Evolutionary Synthesis, Department of Biosciences, University of Oslo, PO Box 1066, Blindern, N-0316 Oslo, Norway.*

*^2^ McDonald Institute for Archaeological Research, Department of Archaeology, University of Cambridge, Downing Street, Cambridge CB2 3ER, United Kingdom.*

Authors for correspondence:

Bastiaan Star, email: bastiaan.star@ibv.uio.no

James H. Barrett, email: jhb41@cam.ac.uk

Sanne Boessenkool, email: sanne.boessenkool@ibv.uio.no

**Proceedings of the Royal Society B**

**Doi: 10.1098/rspb.2018.0978**

**Supplementary Notes**

1. **Generating a Pacific walrus mitochondrial genome sequence 2**
2. **Assessing Restriction Fragment Length Polymorphism in Atlantic walrus 2**
3. **Atlantic walrus control region polymorphisms 3**

**Supplementary Tables 6**

**Supplementary Figures 10**

**Supplementary References 13**

**Supplementary Notes**

1. **Generating a Pacific walrus (*Odobenus rosmarus divergence*) mitochondrial genome sequence**

The publicly released genome sequence of the Pacific walrus [1] includes a mitogenome assembly from Atlantic walrus [2], Genbank ID NC_004029.2. Hence, no *de novo* Pacific walrus mitochondrial reference genome sequence is currently available. To create a Pacific walrus MT genome sequence to root our phylogenetic analyses, we obtained a subset of 169,056,390 paired reads generated for the Pacific walrus genome assembly project (file SRR575502, ENA project ID PRJNA167474, [1]) and aligned these to the Atlantic walrus MT reference genome with PALEOMIX [3] using BWA *mem* v.0.7.5a-r405 [4]. Both collapsed and paired reads were used, resulting in a 13-fold coverage of the mitogenome. The Pacific walrus alignment data and the archaeological Atlantic walrus data were further processed simultaneously.

**2. Assessing Restriction Fragment Length Polymorphism in Atlantic walrus**

Significant population genetic structure based on mtDNA variation in Atlantic walrus has been detected using Restriction Fragment Length Polymorphism (RFLP) analysis targeting the ND1, ND2, and ND3/4 genes [5, 6]. Specifically, several RFLP mtDNA haplotypes have been reported that are solely found in specimens obtained from western Greenland and the Canadian Arctic, and these haplotypes have therefore been suggested to be diagnostic markers, unique to these western populations [5, 6]. We here assess whether the RFLP haplotypes obtained in these earlier studies are linked to the observed monophyletic divergence between the western and eastern clade of Atlantic walrus in our ancient samples. First, we expect diagnostic SNPs supporting the two clades found in the present study to be located in the ND1, ND2 and ND3/ND4 region. Moreover, at least some of these diagnostic SNPs should alter the sequence motif of restriction fragment binding sites specific to the restriction enzymes used in the RFLP studies.

In our mitogenomes, we identify five diagnostic SNPs with principle component weightings greater than 1.25 (smartPCA, EIGENSOFT v.6.1.4 [7]) in ND1 (between 2752 and 3708 bp), three SNPs in ND2 (between 3920 and 4963 bp) and nine SNPs in ND3/ND4 (between 9485 and 11568 bp). In each of these three regions, we find at least one SNP that alters the sequence motif of a restriction enzyme according to each monophyletic clade. In ND1 at position 2982, a G/A polymorphism distorts the GGCCC (western) binding motif of *Sau961* and *HaeIII* into AGCCC (eastern). In ND2 at position 4597, an A/G polymorphism alters the CGACT (eastern) motif restricted by *Hinf1* to CAACT (western). In ND3/ND4 at position 9658, a G/A polymorphism distorts the GGCC (western) binding motif of *HaeIII* into AGCC (eastern). Finally, in ND3/ND4 at position 10616, a T/C polymorphism distorts the TTTAAA (western) binding motif of *Dra1* to ATTAAA (eastern). The haplotype separation obtained by the earlier RFLP studies using these specific restriction enzymes can therefore be explained by diagnostic SNP differentiation that is directly linked to the two monophyletic clades discovered in our ancient mitogenomes.

**3. Atlantic walrus Control Region (CR) Polymorphism**

Within 27 Atlantic walruses sampled from both sides of the Atlantic Ocean, significant population differentiation –identifying a group of monophyletic western Greenland haplotypes– has been detected based on the combined data from the ND1, COI and the mtDNA control region (CR) [8]. Using data from the CR region alone, however, extensive population studies targeting hundreds of individuals show that this region lacks discriminatory power to confidently resolve Atlantic walrus populations within the Northeast Atlantic [9] nor distinguish between western and eastern Atlantic populations [10, 11]. Nevertheless, several haplotypes were found only in the western Greenland region and not in any of the studied populations in East Greenland, Svalbard or Franz Josef land [10, 11]. This observation suggests the existence of CR haplotypes that are only found in walruses in western Greenland. We here associate these CR haplotypes to the monophyletic divergence observed in our historic samples and the 27 modern specimens studied by Lindqvist *et al.* [8].

In our mitogenome data, we identify three SNPs (A/G_15564_, C/T_15760_ and C/T_15779_) with principle component weightings above 1.25 (smartPCA, EIGENSOFT v.6.1.4 [7]) that fall within the region of the CR covered by all earlier studies, between 15328 and 15827 bp [8-11]. In contrast to the diagnostic SNPs in the ND1, ND2, ND3 and ND4 region (see Supplementary Note 2), neither of these CR SNPs *alone* is fixed in either clade, reflecting the lack of power of the CR to differentiate the observed divergence over the entire mitogenome. Yet, we note that the A_15564_ C_15760_ C_15779_ haplotype occurs in 12 out of 15 of the archaeological specimens from the western clade while it is not found in the eastern clade.

We investigate the occurrence of this specific ACC haplotype in 105 publicly available CR sequences (Supplementary Table 3) [8-11] obtained from Genbank. These haplotypes represent over 300 individual Atlantic walruses sampled from multiple locations east and west of Greenland (Figure 1C). For walruses from East Greenland, Svalbard, Frans-Josef land and the Pechora Sea we report the specimen numbers of [9] as this study includes samples analysed by [8]. Historic data from Håøya and Bjørnøya were obtained from [11] and the Northwest Greenland samples were obtained from [8]. From [10] we selected only data from those two locations (Nottingham Island and Hoare Bay) from which more than two specimens were sampled. All obtained sequences were aligned to the Atlantic walrus reference genome using BWA *mem* v.0.7.5a-r405 [4], and their genotypes at position 15564, 15760 and 15779 were scored in IGV [12].

We identify 38 out of 306 modern Atlantic walruses with the same CR haplotype (Supplementary Table 2). None of the modern samples obtained from populations in the Northeast Atlantic contained this ACC haplotype in the CR, several samples in Canada have this haplotype, all samples from Northwest Greenland have this haplotype (Supplementary Table 2, Figure 1C). This result shows that the ACC haplotype we observe in our archaeological mitochondrial data is restricted to modern walrus populations in western Greenland and the Canadian Arctic.

**Supplementary Tables**

**­­Supplementary** **Table 1**. Sample details of archaeological Atlantic walrus specimens. The inferred genetic (*e; eastern clade, w; western clade*) MT lineage of each specimen is also indicated. Based on anatomical representation, animal size, archaeological context and/or MT haplotype all bones except two London specimens can be confidently interpreted as from separate animals. The London specimens could be the left and right alveoli of the same walrus rostrum although they were obtained from different archaeological layers. All samples were taken with museum permission and transported across international borders with the relevant CITES permits.

| Sample number | Museum record | MT  lineage | Modern  location | Discovery  location | Archaeological  site | Date CE | Material | References |
| --- | --- | --- | --- | --- | --- | --- | --- | --- |
|  |  |  |  |  |  |  |  |  |
| 08 | B.1950 | e | NHM, University of Bergen | Moffen, Svalbard | n.a. | c.1850 | Petrous | Anne Karin Hufthammer (a) |
| 09 | JS366 | e | NHM, University of Bergen | Russekeila, Isfjorden, Svalbard | Russekeila | 18th-19th C | Bone | Anne Karin Hufthammer (a) |
| 10 | B.1947 | e | NHM, University of Bergen | Moffen, Svalbard | n.a. | c.1850 | petrous | Anne Karin Hufthammer (a) |
| 11 | B.2036 | e | NHM, University of Bergen | Moffen, Svalbard | n.a. | c.1850 | petrous | Anne Karin Hufthammer (a) |
| 12 | B.1952 | e | NHM, University of Bergen | Moffen, Svalbard | n.a. | c.1850 | petrous | Anne Karin Hufthammer (a) |
| 17 | B.2034 | e | NHM, University of Bergen | Moffen, Svalbard | n.a. | c.1850 | petrous | Anne Karin Hufthammer (a) |
| 21 | B.1877 | e | NHM, University of Bergen | Moffen, Svalbard | n.a. | c.1850 | petrous | Anne Karin Hufthammer (a) |
| 22 | B.1907 | e | NHM, University of Bergen | Moffen, Svalbard | n.a. | c.1850 | petrous | Anne Karin Hufthammer (a) |
| 23 | B.2005 | e | NHM, University of Bergen | Moffen, Svalbard | n.a. | c.1850 | petrous | Anne Karin Hufthammer (a) |
| 24 | B.1973 | e | NHM, University of Bergen | Moffen, Svalbard | n.a. | c.1850 | petrous | Anne Karin Hufthammer (a) |
| 29 | E173:4330 | e | National Museum of Ireland | Dublin | St John's Lane | c.900-1125 | rostrum | Maeve Sikora & Andy Halpin (a) |
| 30 | E173:323 | w | National Museum of Ireland | Dublin | St John's Lane | c.900-1125 | rostrum | Maeve Sikora & Andy Halpin (a) |
| 31 | 21273 | w | MOLA | London | London Guildhall | late 12th-early 13th C | rostrum | (1) |
| 33 | 21274 | w | MOLA | London | London Guildhall | late 12th-early 13th C | rostrum | (1) |
| 34 | E172:4311 | e | National Museum of Ireland | Dublin | Fishamble Street II | 11th C | rostrum | (2); Maeve Sikora & Andy Halpin (a) |
| 38 | N10102 | e | NTNU Museum of Natural History and Archaeology | Trondheim | Søndre gate Felt II,  Felt T | 1111 or shortly before | rostrum | Axel Christophersen (a) |
| 39 | BRM0/46092/1 | w | KHM, University of Bergen | Bergen | BRM0 | 1120-1170 | rostrum | (3) |
| 41 | N32091 | w | NTNU Museum of Natural History and Archaeology | Trondheim | Folkebibliotekstomten | 1225-1275 | rostrum | Axel Christophersen (a) |
| 42 | BRM4/4176/1 | w | KHM, University of Bergen | Bergen | BRM4 | late 12th C  to 1225 | rostrum | (3) |
| 43 | BRM0/2709/1 | e | KHM, University of Bergen | Bergen | BRM0 | 1248-1332 | rostrum | Gitte Hansen (a) |
| 44 | N29494 | w | NTNU Museum of Natural History and Archaeology | Trondheim | Folkebibliotekstomten | 1150-1175 | rostrum | Axel Christophersen (a) |
| 45 | BRM0/87411/1 | w | KHM, University of Bergen | Bergen | BRM0 | 1248-1332 | rostrum | Gitte Hansen (a) |
| 46 | N167587 | w | NTNU Museum of Natural History and Archaeology | Trondheim | Archbishop's Palace | 1500-1532 | rostrum | Sæbjørg Nordeide (a) |
| 47 | N37603 | e | NTNU Museum of Natural History and Archaeology | Trondheim | Folkebibliotekstomten | post 1600 | rostrum | Axel Christophersen (a) |
| 48 | N203145 | w | NTNU Museum of Natural History and Archaeology | Trondheim | Kjøpmannsgata,  Felt 93/2 | n.a. | rostrum | Axel Christophersen (a) |
| 49 | F51873 | w | NIKU, Oslo | Oslo | Follo D1 Vest | 1250-1350 | rostrum | Lars Morten Fuglevik (a) |
| 62 | C23798 | e | KHM, University of Oslo | Oslo | Ladegårds 1903 | n.a. | rostrum | (4); Marianne Vedeler (a) |
| 63 | 8240a | e | Sigtuna Museum | Sigtuna | kv Urmakaren | 1000-1050 | tusk offcut | Anders Söderberg (a) |
| 64 | 8241a | e | Sigtuna Museum | Sigtuna | kv Urmakaren | 1000-1050 | tusk offcut | Anders Söderberg (a) |
| 65 | 8241b | e | Sigtuna Museum | Sigtuna | kv Urmakaren | 1000-1050 | tusk offcut | Anders Söderberg (a) |
| 67 | FNR 29257 | w | Sigtuna Museum | Sigtuna | kv Trädgårdsmästaren | 1200-1230 | rostrum | (5); Anders Söderberg (a) |
| 68 | n.a. | e | Schleswig-Holsteinische Landesmuseen Schloss Gottorf | Schleswig | Hafenstraße 13 | 12th-13th C | rostrum | (6); Volker Hilberg (a) |
| 69­­­ | P151/2017 KMG | e | Natural History Museum of Denmark | Igaliku (Gardar), Greenland | Kirkegaarden 1926 | late 10th-  12th C | rostrum | (7); Jette Arneborg (a); Kristian Murphy Gregersen (a) |
| 70 | P149/2017 KMG | e | Natural History Museum of Denmark | Igaliku (Gardar), Greenland | Kirkegaarden 1926 | late 10th-  12th C | rostrum | (7); Jette Arneborg (a); Kristian Murphy Gregersen (a) |
| 71 | P153/2017 KMG | w | National Museum of Denmark | Igaliku (Gardar), Greenland | na | late 10th-  12th C | rostrum | (7); Jette Arneborg (a); Kristian Murphy Gregersen (a) |
| 72 | P155/2017 KMG | w | National Museum of Denmark | Igaliku (Gardar), Greenland | na | late 10th-  12th C | rostrum | (7); Jette Arneborg (a); Kristian Murphy Gregersen (a) |
| 73 | MHNLM 2004.3.53 | w | Musée Vert, muséum d'histoire naturelle du Mans | Na | na | 13th-14th C | postcanine tooth root | (8,9); Nicolas Morel (a) |

(a) Personal communication­.

References:

1. Bowsher D., Dyson T., Holder N., Howell I. 2007 *The London Guildhall: An Archaeological History of a Neighbourhood from Early Medieval to Modern Times*. London, Museum of London Archaeology Service.

2. Caulfield D. 1992 Walrus skull and tusk fragment. In *From Viking to Crusader: Scandinavia and Europe 800-1200* (eds. Roesdahl E., Wilson D.W.), p. 385. Uddevalla, Sweden, Nordic Council.

3. Hansen G. 2005 *Bergen c.800-c.1170: The emergence of a town*. Bergen, Fagbokforlaget as.

4. Grieg S. 1933 *Middelalderske Byfund fra Bergen og Oslo*. Oslo, A.W. Brøggers Boktrykkeri A/S.

5. Karlsson J. 2016 *Spill om Djur, Hantverk och Nätverk i Mälarområdet under Vikingatid och Medeltid*. Stockholm, Institutionen för arkeologi och antikens kultur, Stockholms universitet.

6. Rösch F. 2015 Das Schleswiger Hafenviertel im Hochmittelalter: Entstehung – Entwicklung – Topographie. Teil II: Abbildungen, Tabellen, Katalog I [PhD]. Kiel, Christian-Albrechts-Universität zu Kiel.

7. Degerbøl M. 1929 Animal bones from the Norse Ruins at Gardar. In *Norse Ruins at Gardar: The Episcopal Seat of Medieval Greenland* (eds. Nørlund P., Roussell A.), pp. 183-192. Copenhagen, The Commission for Scientific Research in Greenland.

8. Roesdahl E., Stoklund M. 2006 Un crâne de morse décoré et gravé de runes: Á propos d'une découverte récente dans un musée du Mans. *Proxima Thulé* **5**, 9-38.

9. Imer L.M. 2017 *Peasants and Prayers: The Inscriptions of Norse Greenland*. Copenhagen, University Press of Southern Denmark.

**Supplementary Table 2.** Sequencing details of 37 archaeological Atlantic walrus samples. Estimates for library clonality and endogenous DNA content were obtained by aligning reads to the nuclear Pacific walrus reference genome [1]. To obtain mitochondrial (MT) data, reads were aligned separately to the Atlantic walrus mitogenome [2].

| Sample ID | Reads  (millions) | Clonality (%) | Endogenous DNA (fraction) | MT fold coverage | Insert length(bp) |
| --- | --- | --- | --- | --- | --- |
| 08 | 13 | 0.01 | 0.44 | 43 | 70 |
| 09 | 16 | 0.01 | 0.09 | 17 | 80 |
| 10 | 19 | 0.01 | 0.33 | 59 | 79 |
| 11 | 6 | 0.00 | 0.23 | 8 | 62 |
| 12 | 5 | 0.00 | 0.43 | 30 | 68 |
| 17 | 20 | 0.01 | 0.68 | 125 | 78 |
| 21 | 15 | 0.01 | 0.71 | 80 | 91 |
| 22 | 15 | 0.01 | 0.67 | 89 | 87 |
| 23 | 12 | 0.01 | 0.35 | 73 | 96 |
| 24 | 16 | 0.01 | 0.69 | 49 | 81 |
| 29 | 12 | 0.02 | 0.29 | 52 | 69 |
| 30 | 17 | 0.03 | 0.17 | 74 | 72 |
| 31 | 9 | 0.02 | 0.16 | 81 | 72 |
| 33 | 17 | 0.03 | 0.01 | 8 | 94 |
| 34 | 30 | 0.42 | 0.02 | 7 | 58 |
| 38 | 5 | 0.04 | 0.35 | 16 | 76 |
| 39 | 8 | 0.03 | 0.01 | 13 | 61 |
| 41 | 1 | 0.01 | 0.48 | 10 | 70 |
| 42 | 11 | 0.02 | 0.01 | 13 | 74 |
| 43 | 13 | 0.08 | 0.00 | 9 | 71 |
| 44 | 10 | 0.01 | 0.49 | 14 | 80 |
| 45 | 12 | 0.02 | 0.11 | 24 | 75 |
| 46 | 10 | 0.03 | 0.03 | 22 | 97 |
| 47 | 11 | 0.05 | 0.30 | 9 | 64 |
| 48 | 11 | 0.03 | 0.42 | 179 | 79 |
| 49 | 12 | 0.02 | 0.02 | 9 | 79 |
| 62 | 15 | 0.06 | 0.49 | 295 | 62 |
| 63 | 12 | 0.01 | 0.06 | 46 | 77 |
| 64 | 9 | 0.01 | 0.03 | 14 | 72 |
| 65 | 12 | 0.02 | 0.37 | 438 | 73 |
| 67 | 6 | 0.01 | 0.08 | 13 | 69 |
| 68 | 10 | 0.02 | 0.19 | 104 | 67 |
| 69 | 23 | 0.01 | 0.01 | 5 | 76 |
| 70 | 27 | 0.01 | 0.02 | 10 | 72 |
| 71 | 27 | 0.01 | 0.01 | 6 | 69 |
| 72 | 35 | 0.01 | 0.02 | 10 | 69 |
| 73 | 16 | 0.01 | 0.15 | 68 | 72 |

**Supplementary Table 3.** Genotypes for three control region SNPs in 105 Atlantic walrus control region sequences. These SNPs are located between 15328 and 15827 bp. For each of the sequences, we show their Genbank accession number, study from which the data was obtained, and whether the individuals with these sequences originated from western Greenland or Canada (West) or the Northeast Atlantic (East)*.* The MT location and alleles (between brackets) for each SNP are given. The A_15564_ C_15760_C_15779_ haplotype that only occurs in western Greenland and the Canadian Arctic is highlighted (**bold**).

|  |  | Location | SNP Genotype | | |
| --- | --- | --- | --- | --- | --- |
| GenBank Accession | Study |  | 15564 (A/G) | 15760 (C/T) | 15779 (C/T) |
| KJ522887.1 | McLoad 2014 | West | A | T | C |
| KJ522888.1 | McLoad 2014 | West | A | T | C |
| KJ522889.1 | McLoad 2014 | West | A | T | C |
| KJ522890.1 | McLoad 2014 | West | G | T | T |
| KJ522891.1 | McLoad 2014 | West | A | T | C |
| KJ522892.1 | McLoad 2014 | West | A | T | C |
| KJ522893.1 | McLoad 2014 | West | G | T | T |
| KJ522894.1 | McLoad 2014 | West | A | T | C |
| KJ522895.1 | McLoad 2014 | West | G | T | C |
| **KJ522896.1** | **McLoad 2014** | **West** | **A** | **C** | **C** |
| KJ522897.1 | McLoad 2014 | West | A | T | C |
| **KJ522898.1** | **McLoad 2014** | **West** | **A** | **C** | **C** |
| KJ522899.1 | McLoad 2014 | West | G | T | T |
| KJ522900.1 | McLoad 2014 | West | G | T | T |
| KJ522901.1 | McLoad 2014 | West | G | T | T |
| **KJ522902.1** | **McLoad 2014** | **West** | **A** | **C** | **C** |
| **KJ522903.1** | **McLoad 2014** | **West** | **A** | **C** | **C** |
| **KJ522904.1** | **McLoad 2014** | **West** | **A** | **C** | **C** |
| KJ522905.1 | McLoad 2014 | West | A | T | C |
| KJ522906.1 | McLoad 2014 | West | G | T | C |
| KJ522907.1 | McLoad 2014 | West | A | T | C |
| KJ522908.1 | McLoad 2014 | West | A | T | C |
| KJ522909.1 | McLoad 2014 | West | A | T | C |
| KJ522910.1 | McLoad 2014 | West | G | T | T |
| **KJ522911.1** | **McLoad 2014** | **West** | **A** | **C** | **C** |
| KJ522912.1 | McLoad 2014 | West | G | T | C |
| KJ522913.1 | McLoad 2014 | West | A | T | C |
| KJ522914.1 | McLoad 2014 | West | A | T | C |
| KJ522915.1 | McLoad 2014 | West | A | T | C |
| KJ522916.1 | McLoad 2014 | West | A | T | C |
| KJ522917.1 | McLoad 2014 | West | A | T | C |
| KJ522918.1 | McLoad 2014 | West | A | T | C |
| KJ522919.1 | McLoad 2014 | West | A | T | C |
| **KJ522920.1** | **McLoad 2014** | **West** | **A** | **C** | **C** |
| **KJ522921.1** | **McLoad 2014** | **West** | **A** | **C** | **C** |
| **KJ522922.1** | **McLoad 2014** | **West** | **A** | **C** | **C** |
| MF166700.1 | Andersen 2017 | East | G | T | C |
| MF166701.1 | Andersen 2017 | East | A | T | T |
| MF166702.1 | Andersen 2017 | East | G | T | C |
| MF166703.1 | Andersen 2017 | East | G | T | C |
| MF166704.1 | Andersen 2017 | East | A | T | T |
| MF166705.1 | Andersen 2017 | East | G | T | T |
| MF166706.1 | Andersen 2017 | East | G | T | T |
| MF166707.1 | Andersen 2017 | East | A | T | C |
| MF166708.1 | Andersen 2017 | East | A | C | T |
| MF166709.1 | Andersen 2017 | East | G | T | C |
| MF166710.1 | Andersen 2017 | East | G | T | T |
| MF166711.1 | Andersen 2017 | East | G | T | C |
| MF166712.1 | Andersen 2017 | East | A | C | T |
| MF166713.1 | Andersen 2017 | East | A | T | C |
| MF166714.1 | Andersen 2017 | East | A | T | T |
| MF166715.1 | Andersen 2017 | East | G | T | C |
| MF166716.1 | Andersen 2017 | East | G | T | C |
| MF166717.1 | Andersen 2017 | East | G | T | T |
| MF166718.1 | Andersen 2017 | East | A | T | T |
| MF166719.1 | Andersen 2017 | East | G | T | T |
| MF166720.1 | Andersen 2017 | East | A | T | T |
| MF166721.1 | Andersen 2017 | East | A | T | T |
| MF166722.1 | Andersen 2017 | East | G | T | T |
| MF166723.1 | Andersen 2017 | East | A | T | T |
| MF166724.1 | Andersen 2017 | East | A | T | T |
| KU710183.1 | Bachman 2016 | East | A | T | T |
| KU710184.1 | Bachman 2016 | East | G | T | T |
| KU710185.1 | Bachman 2016 | East | G | T | C |
| KU710186.1 | Bachman 2016 | East | A | T | T |
| KU710187.1 | Bachman 2016 | East | G | T | C |
| KU710189.1 | Bachman 2016 | East | A | T | T |
| KU710190.1 | Bachman 2016 | East | A | T | T |
| KU710191.1 | Bachman 2016 | East | A | T | T |
| KU710192.1 | Bachman 2016 | East | G | T | T |
| KU710193.1 | Bachman 2016 | East | G | T | C |
| KU710194.1 | Bachman 2016 | East | G | T | T |
| KU710195.1 | Bachman 2016 | East | G | T | T |
| KU710196.1 | Bachman 2016 | East | G | T | T |
| KU710197.1 | Bachman 2016 | East | A | T | T |
| KU710198.1 | Bachman 2016 | East | A | T | T |
| KU710199.1 | Bachman 2016 | East | G | T | C |
| KU710200.1 | Bachman 2016 | East | G | T | T |
| EU728544.1 | Lindquist 2009 | East | A | T | T |
| EU728545.1 | Lindquist 2009 | East | A | T | T |
| EU728546.1 | Lindquist 2009 | East | A | T | T |
| EU728547.1 | Lindquist 2009 | East | A | T | C |
| EU728548.1 | Lindquist 2009 | East | A | T | T |
| **EU728549.1** | **Lindquist 2009** | **West** | **A** | **C** | **C** |
| **EU728550.1** | **Lindquist 2009** | **West** | **A** | **C** | **C** |
| **EU728551.1** | **Lindquist 2009** | **West** | **A** | **C** | **C** |
| **EU728552.1** | **Lindquist 2009** | **West** | **A** | **C** | **C** |
| **EU728553.1** | **Lindquist 2009** | **West** | **A** | **C** | **C** |
| EU728554.1 | Lindquist 2009 | East | A | T | T |
| EU728555.1 | Lindquist 2009 | East | A | T | T |
| EU728556.1 | Lindquist 2009 | East | A | T | T |
| EU728557.1 | Lindquist 2009 | East | A | T | T |
| EU728558.1 | Lindquist 2009 | East | A | T | T |
| **EU728559.1** | **Lindquist 2009** | **West** | **A** | **C** | **C** |
| **EU728560.1** | **Lindquist 2009** | **West** | **A** | **C** | **C** |
| **EU728561.1** | **Lindquist 2009** | **West** | **A** | **C** | **C** |
| EU728565.1 | Lindquist 2009 | East | A | C | T |
| EU728566.1 | Lindquist 2009 | East | A | T | T |
| EU728567.1 | Lindquist 2009 | East | A | T | T |
| EU728568.1 | Lindquist 2009 | East | A | T | C |
| EU728569.1 | Lindquist 2009 | East | G | T | T |
| EU728570.1 | Lindquist 2009 | East | G | C | T |
| EU728571.1 | Lindquist 2009 | East | G | T | C |
| EU728572.1 | Lindquist 2009 | East | G | T | C |
| EU728573.1 | Lindquist 2009 | East | G | T | C |

**Supplementary Figures**

**
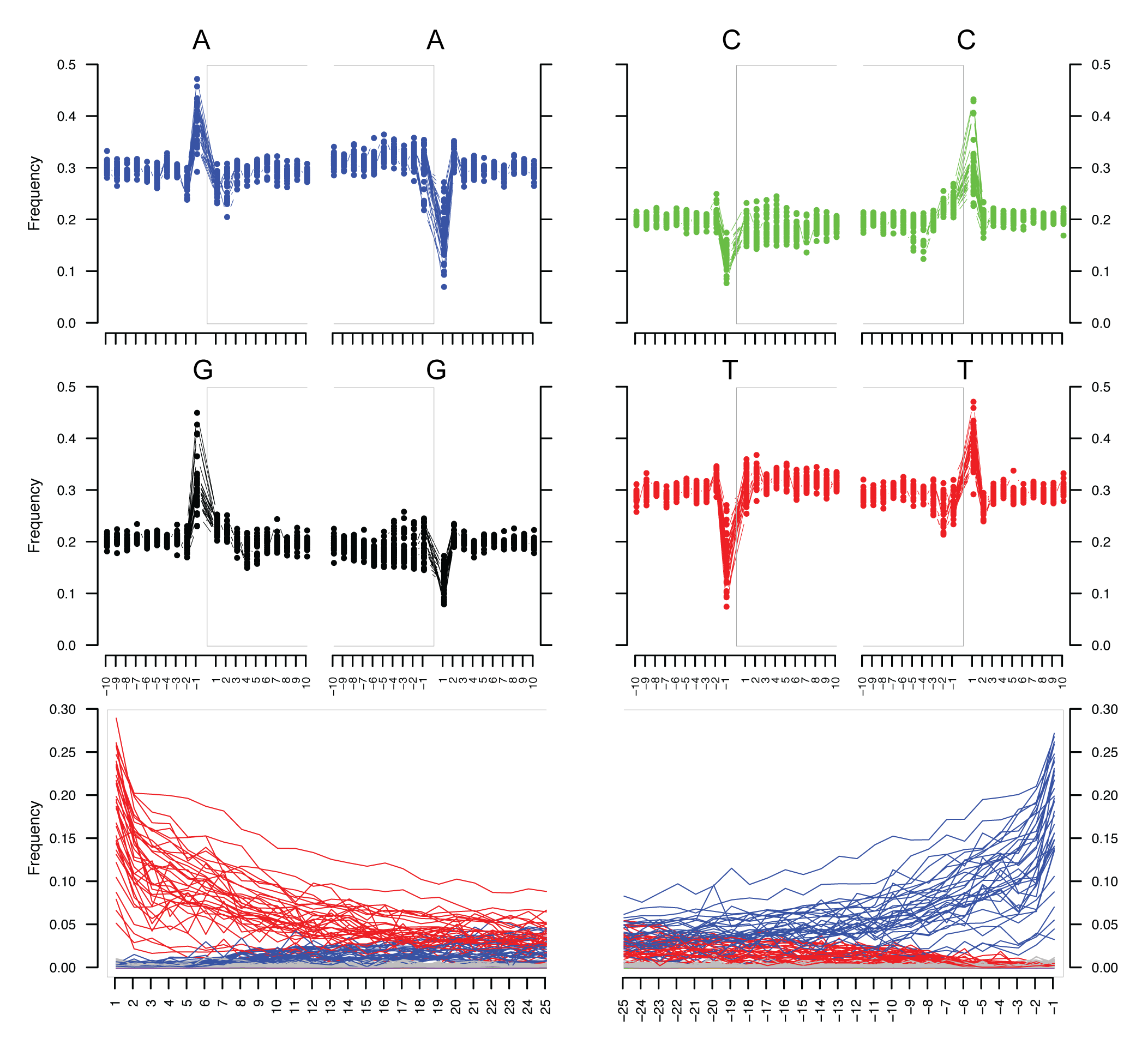
**

**Supplementary Figure 1.** aDNA fragmentation and mis-incorporation patterns of sequencing read data from 37 arch­aeological Atlantic walrus samples. All samples show the typical fragmentation (top four panels), elevated 5'-end C->T (bottom left panel) and elevated 3'-end G->A substitution patterns (bottom right panel) expected from sequencing authentic aDNA data. Patterns were obtained using MapDamage v. 2.0.6 after down-sampling BAM files to 1,000,000 reads if applicable.


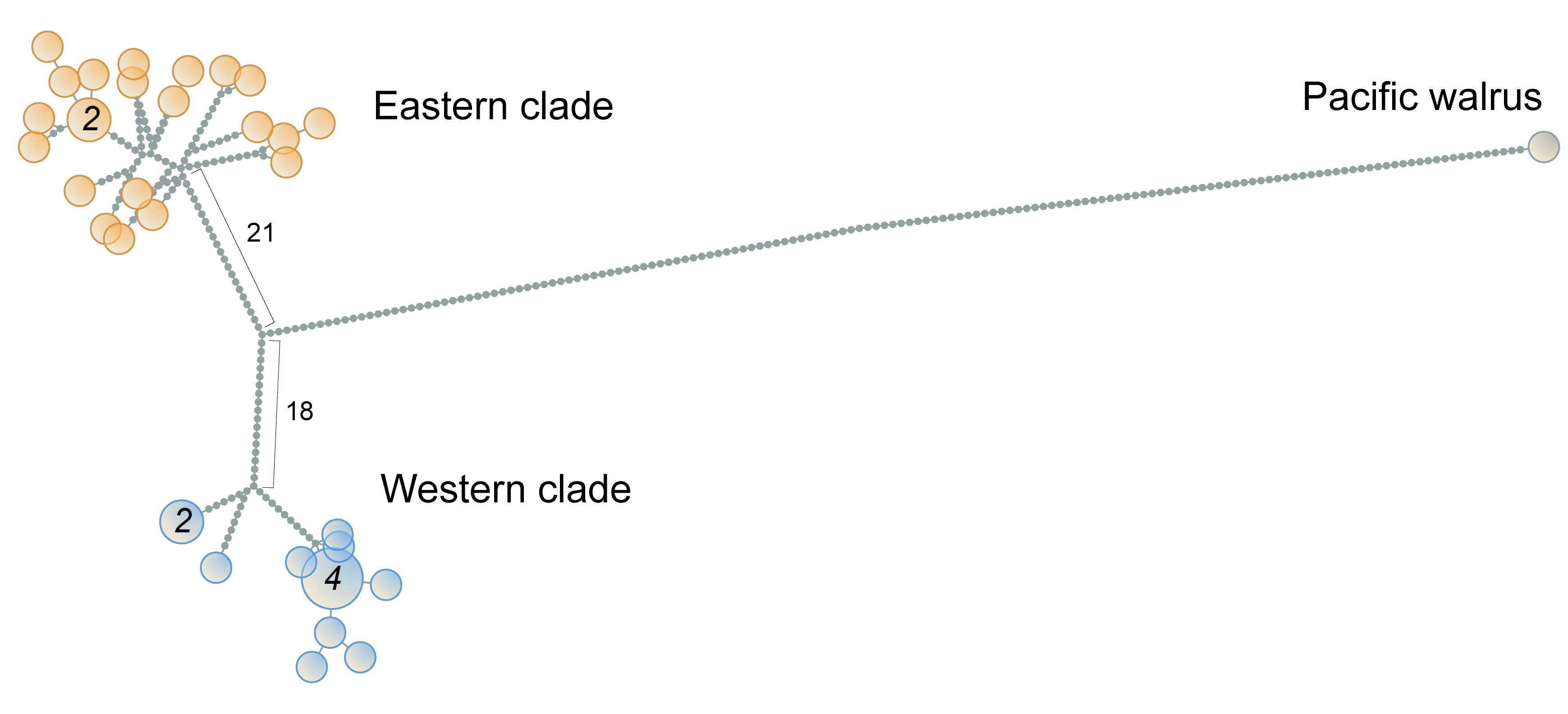


**Supplementary Figure 2.** Haplotype genealogy graph of 37 archaeological Atlantic walruses and one Pacific walrus. Haplotypes belonging to the Western clade (*blue*), Eastern clade (*orange*) or the Pacific walrus (*grey*) are separated by a number of substitutions (grey edges indicated with a number for the two main Atlantic walrus branches). Circle size reflects the number of specimens with an identical haplotype, and where this is >1 the number is specified within the circle.


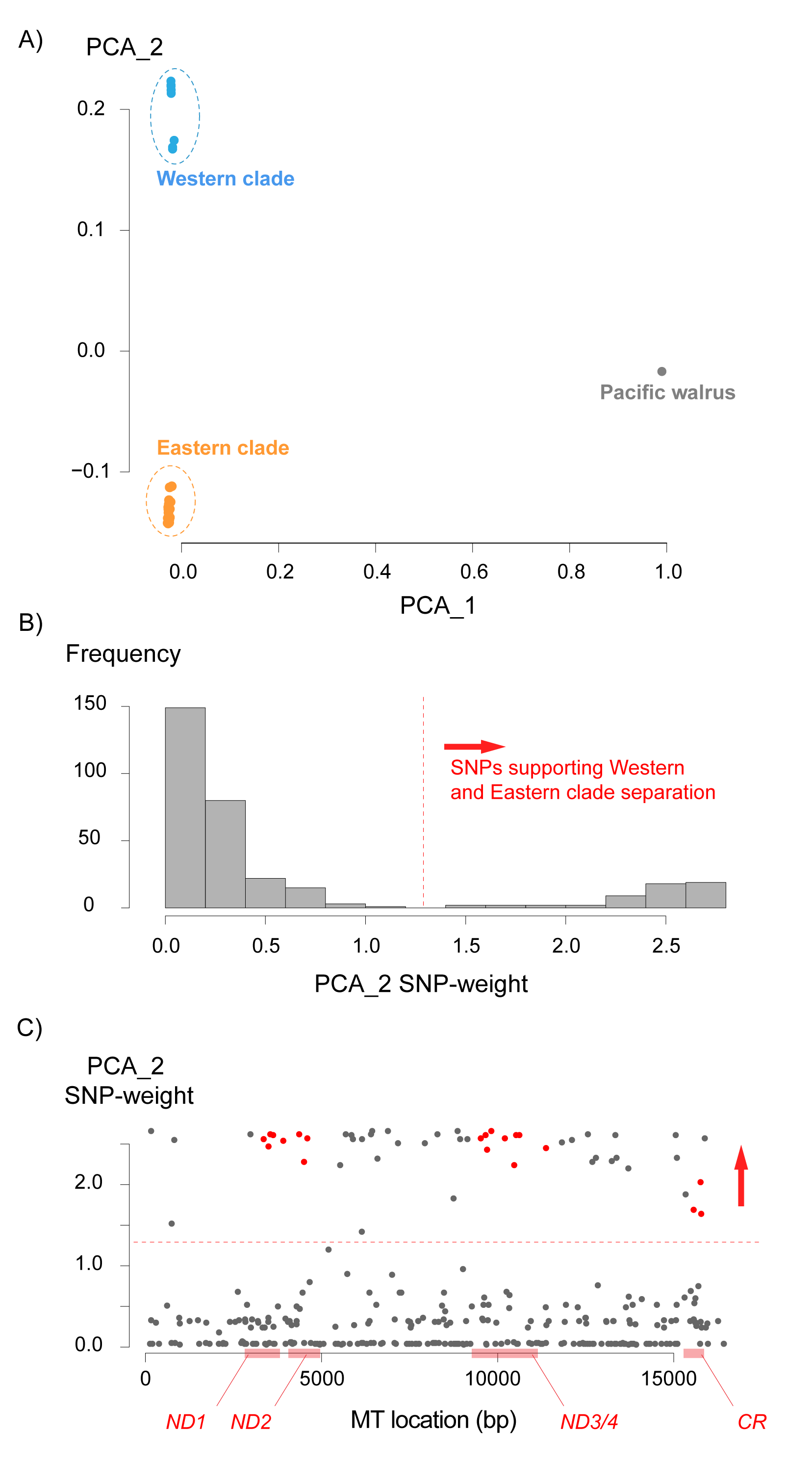
­

**Supplementary Figure 3.** Genetic population structure based on whole mitochondrial genome data in 37 archaeological Atlantic walruses and one Pacific walrus. ***(a)*** Principle component analysis (PCA) based on 346 SNPs using smartPCA, EIGENSOFT v.6.1.4. The first principle component significantly (eigenvalue = 15.5, Tracy-Widom (TW) stat = 1.76, *p*-value = 0.01) differentiates the Pacific walrus (*grey*) from the Atlantic specimens. The second principle component significantly (eigenvalue = 7.01, TW stat = 4.26, *p*-value = 0.0001) differentiates the western (*blue*) and eastern (*orange*) Atlantic walrus clades. ***(b)*** A bimodal distribution characterizes the SNP-weightings of the second Principle Component. SNPs with a weighting above 1.25 support the western and eastern differentiation, and those with the highest values are exclusively associated with either clade. ***(c)*** SNPs supporting the western and eastern differentiation are located throughout the mitochondrial genome with a subset of SNPs (*red*) located in those regions (*ND1*, *ND2*, *ND3*/*4* and the control region *CR, red italic*) investigated in previous studies.

**Supplementary References**

1. Foote A.D., Liu Y., Thomas G.W.C., Vinar T., Alfoldi J., Deng J., Dugan S., van Elk C.E., Hunter M.E., Joshi V., et al. 2015 Convergent evolution of the genomes of marine mammals. *Nat Genet* **47**(3), 272-275. (doi:10.1038/ng.3198).

2. Arnason U., Adegoke J.A., Bodin K., Born E.W., Esa Y.B., Gullberg A., Nilsson M., Short R.V., Xu X., Janke A. 2002 Mammalian mitogenomic relationships and the root of the eutherian tree. *Proc Natl Acad Sci USA* **99**(12), 8151-8156. (doi:10.1073/pnas.102164299).

3. Schubert M., Ermini L., Der Sarkissian C., Jónsson H., Ginolhac A., Schaefer R., Martin M.D., Fernández R., Kircher M., McCue M. 2014 Characterization of ancient and modern genomes by SNP detection and phylogenomic and metagenomic analysis using PALEOMIX. *Nat Protoc* **9**(5), 1056-1082. (doi:10.1038/nprot.2014.063).

4. Li H., Durbin R. 2009 Fast and accurate short read alignment with Burrows-Wheeler transform. *Bioinformatics* **25**(14), 1754-1760. (doi:10.1093/bioinformatics/btp324).

5. Andersen L.W., Born E.W., Gjertz I., Wiig Ø., Holm L.E., Bendixen C. 1998 Population structure and gene flow of the Atlantic walrus (Odobenus rosmarus rosmarus) in the eastern Atlantic Arctic based on mitochondrial DNA and microsatellite variation. *Mol Ecol* **7**(10), 1323-1336. (doi:10.1046/j.1365-294x.1998.00455.x).

6. Born E., Andersen L., Gjertz I., Wiig Ø. 2001 A review of the genetic relationships of Atlantic walrus (Odobenus rosmarus rosmarus) east and west of Greenland. *Polar Biology* **24**(10), 713-718. (doi:10.1007/s003000100).

7. Patterson N., Price A.L., Reich D. 2006 Population structure and eigenanalysis. *PLoS genet* **2**(12), e190. (doi:10.1371/journal.pgen.0020190).

8. Lindqvist C., Bachmann L., Andersen L.W., Born E.W., Arnason U., Kovacs K.M., Lydersen C., Abramov A.V., Wiig Ø. 2009 The Laptev Sea walrus Odobenus rosmarus laptevi: an enigma revisited. *Zoologica Scripta* **38**(2), 113-127. (doi:10.1111/j.1463‐6409.2008.00364.x).

9. Andersen L.W., Jacobsen M.W., Lydersen C., Semenova V., Boltunov A., Born E.W., Wiig Ø., Kovacs K.M. 2017 Walruses (Odobenus rosmarus rosmarus) in the Pechora Sea in the context of contemporary population structure of Northeast Atlantic walruses. *Biological Journal of the Linnean Society* **122**(4), 897-915. (doi:10.1093/biolinnean/blx093).

10. McLeod B.A., Frasier T.R., Lucas Z. 2014 Assessment of the extirpated Maritimes walrus using morphological and ancient DNA analysis. *PLoS ONE* **9**(6), e99569. (doi:10.1371/journal.pone.0099569).

11. Lindqvist C., Roy T., Lydersen C., Kovacs K.M., Aars J., Wiig Ø., Bachmann L. 2016 Genetic diversity of historical Atlantic walruses (Odobenus rosmarus rosmarus) from Bjørnøya and Håøya (Tusenøyane), Svalbard, Norway. *BMC research notes* **9**(1), 112. (doi:10.1186/s13104-016-1907-8).

12. Robinson J.T., Thorvaldsdóttir H., Winckler W., Guttman M., Lander E.S., Getz G., Mesirov J.P. 2011 Integrative genomics viewer. *Nature Biotechnology* **29**, 24. (doi:10.1038/nbt.1754).
